# Inter-observer variation in two-dimensional and three-dimensional ultrasound measurement of papillary thyroid microcarcinoma

**DOI:** 10.1186/s40644-023-00613-3

**Published:** 2023-10-05

**Authors:** Lin Yan, Ling Ren, Yingying Li, Yukun Luo

**Affiliations:** Department of Ultrasound, the First Medical Centre, Chinese PLA General Hospital, No.28 Fuxing Road, Haidian District, Beijing, 100853 China

**Keywords:** Thyroid cancer, Papillary, Ultrasound, Reproducibility of results

## Abstract

**Backgrounds:**

The reliable ultrasound (US) measurements of papillary thyroid microcarcinoma (PTMC) are very important during active surveillance. This prospective study was design to investigate the inter-observer reliability and agreement of two- dimensional ultrasound(2DUS) and three-dimensional ultrasound(3DUS) in the measurement of maximum diameter and volume for PTMC.

**Methods:**

This prospective study included 51 consecutive patients with solitary PTMC confirmed by biopsy. Two independent observers performed measurements of each tumor using a standardized measurement protocol. The maximum diameter was the largest one of the three diameters measured on the largest transverse and longitudinal 2DUS images. 2DUS volume was calculated using ellipsoid formula method. The virtual organ computer aided analysis(VOCAL) was used to determine 3DUS volume. The inter-observer reliability was assessed using intraclass correlation coefficient(ICC) with 95% confidence intervals(CIs). Bland-Altman analysis was used to evaluate agreement, and expressed as a bias with 95% limits of agreement(LOA).

**Results:**

The maximum diameter was 0.78 ± 0.14 cm. Volume measured by 3DUS was significantly smaller than that by 2DUS(0.163 ± 0.074 cm^3^ vs. 0.175 ± 0.078 cm^3^, *P* = 0.005). The ICCs of inter-observer reliability of maximum diameter, 2DUS volume and 3DUS volume was 0.922(0.864–0.955), 0.928(0.874–0.959), and 0.974(0.955–0.985), respectively. The ICCs of 2DUS and 3DUS volume was 0.955(0.909–0.976). The inter-observer agreement of maximum diameter, 2DUS volume and 3DUS volume was 1.096(0.7322 to 1.459), 1.008(0.5802–1.435), and 1.011(0.7576–1.265), respectively. The inter-observer agreement of 2DUS and 3DUS volume was 1.096(0.7322 to 1.459).

**Conclusion:**

Maximum diameter had the lowest degree of observer variation among all the measurements. Volume measured by 3DUS had lower variability and higher repeatability than that by 2DUS, which might be helpful to provide more reliable estimates of tumor size for PTMC.

**Supplementary Information:**

The online version contains supplementary material available at 10.1186/s40644-023-00613-3.

## Introduction

The global incidence of papillary thyroid microcarcinoma(PTMC) has risen substantially worldwide in the past three decades [1, 2], following the widespread adoption of ultrasound(US) and other diagnostic imaging modalities [3]. Because mot PTMC are low risk with an excellent prognosis, the optimal management remains controversial [4].

To avoid overtreatment, active surveillance(AS) has been recommended as a new management option to immediate surgery for adult patients with biopsy-proven low-risk PTMC [3, 5], and has favorable results [6–10] Conventional two-dimensional ultrasound(2DUS) was the most widely used imaging modality in the routine follow-up of AS. When the appearance of new tumors and/or lymph node metastasis(LNM), or tumor size enlargement were found, conversion surgery was recommended [5]. The tumor size enlargement was initially defined as an increase in maximal diameter by more than 3.0 mm [8, 11]. Recently, some studies also defined enlargement as a 50% increase in tumor volume [12–14]. However, the quality of US evaluation was limited by the observer variation, which posed a challenge for the implementation of this management in real-world practice [14–16].

Understanding of the inter-observer variations of US was necessary for accurate evaluation [17, 18]. A previous study reported the inter-observer variation of maximum diameter and volume of PTMC was from − 26.6 to 24.5%, and from − 65.8 to 64.4%, respectively [19]. With the advent and progress, three-dimensional ultrasound(3DUS) could scan the target organs or lesions by a single sweep of an US beam and provide the images in multiple slices and planes, which has been applied for fetal growth, tumor diagnosis and interventional therapy [20–23]. It was reported that 3DUS could overcome the drawbacks of 2DUS, making the US examination more objective and less observer dependent, especially in the field of volumetry [24–29]. To the best of our knowledge, little is known of the inter-observer variations of 2DUS and 3DUS in the measurement of PTMC.

Therefore, the purpose of this prospective study was to investigate the inter-observer variations of 2DUS and 3DUS in the measurement of maximum diameter and volume for PTMC.

## Methods

### Study design

This prospective study was approved by the Institutional Review Board of our hospital(S2020-237-01). All the enrolled patients fulfilled these inclusion criteria: [1] confirmation of solitary PTMC by fine-needle aspiration (FNA) or core-needle biopsy(CNB); [2] serum thyroid hormone and thyrotropin levels within normal ranges; [3] accept two complete sets of evaluation, including 2DUS and 3DUS by two observers. Exclusion criteria were: [1] benign results or follicular neoplasm on FNA or CNB; [2] patients with a history of neck irradiation or thyroid disease treatment; [3] patients with neck extension disorder that could not tolerate two complete sets of US scans by two observers.

The sample size was calculated by PASS 15 software (NCSS LCC., Kaysville, UT, USA). The type 1 error was 0.05, and the power was 0.8 based on a two-sided effect. A sample size of 50 subjects with two observers per subject was needed to detect an intraclass correlation coefficient (ICC) of 0.95 by the two modalities in the measurement of volume when the null hypothesis one was 0.9. Therefore, between Jan 2021 to March 2021, this study recruited 51 consecutive patients with solitary PTMC who underwent 2DUS and 3DUS evaluation.

### Measurement

Two physicians (Observer A with more than 10-year experience in thyroid US; Observer B with 5-year experience in thyroid US) performed all the measurements using a SAMSUNG RS85A instrument (SAMSUNG) equipped with an internal 3DUS virtual organ computer aided analysis (VOCAL) program. A 3–12 MHz linear array transducer (L3-12 A) was used to acquire 2DUS images, and a 3–14 MHz volume transducer (LV3-14) was used for 3DUS images acquisition. The thyroid parenchyma background status was defined as normal or Hashimoto thyroiditis [thyroid peroxidase antibody (TPOAb > 60 IU/mL) with or without anti-thyroglobulin antibodies (TgAb > 60 IU/mL)].

**Fig. 1:**
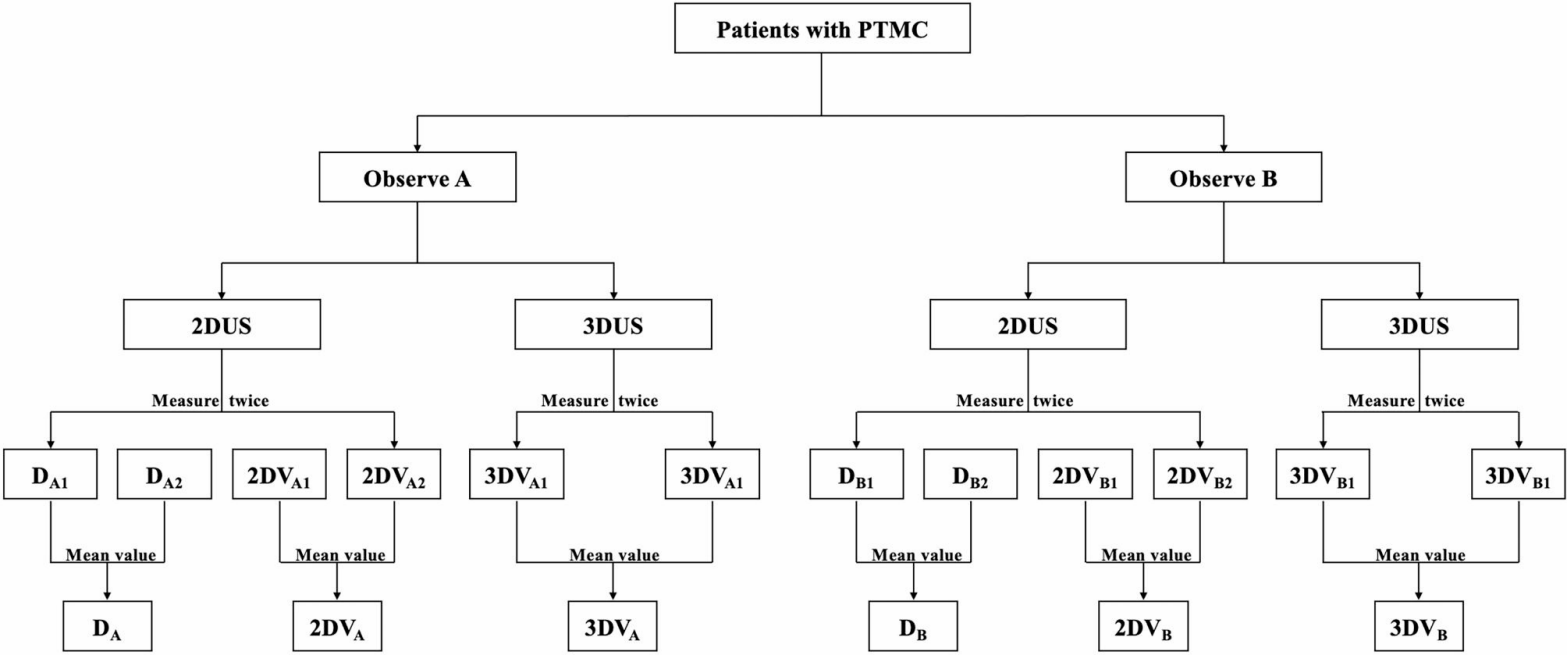
Measurement flowchart. 2DUS: two-dimensional ultrasound; 3DUS: three-dimensional ultrasound; D: maximum diameter; 2DV: volume measured by two-dimensional ultrasound; 3DV: volume measured by three-dimensional ultrasound

Prior to the study, the two observers underwent a training session that consisted of 20 unenrolled cases to acquaint themselves with 3D scanning and manually outlined. To obtain objective measurement, the two observers standardized a measurement protocol as follows(Fig. 1):

(1) Patients were scanned consecutively by the two observers. Only one observer was present in the exanimation room at any time. For each patient, each observer performed a complete new set of scans for the measurement, consisting of 2DUS and 3DUS, without knowledge of the other physician’s results.

(2) During the examination, 2DUS was performed first. For each tumor, the location, composition, echogenicity, shape, margin and echogenic foci were evaluated [30]. The anteroposterior and transverse diameters of tumor were measured on the transverse US image with the largest dimensions, and the longitudinal diameter was measured on the longitudinal US image with the largest dimensions. Tumor was measured with the calipers placed outside of any visible halo [31]. All the measurements were made to the nearest 0.01 cm. After measuring the three diameters, the largest one was defined as the maximum diameter. The 2DUS volume of tumor was calculated using the ellipsoid formula methods as follows: V = πabc/6 (V is the 2DUS volume, while a is the longitudinal diameter, b and c are anteroposterior and transverse diameters, π is 3.1415). The three diameters were measured twice to obtain the means of maximum diameter and 2DUS volume by each observer.

(3) When 2DUS examinations were finished, 3DUS mode was activated to view the largest longitudinal image of tumor. For the 3D data acquisition, the entire tumor was scanned through a single sweep. Each observer scanned the tumor twice and the 3DUS images was stored in the hard disk of the system for further analysis. The images were reviewed and measured in the same after all examinations were completed. The VOCAL method was used to reconstruct and postprocess the 3DUS images to calculate the volume. With 3 orthogonal slices simultaneously displayed, the longitudinal US image plane was selected as A plane. Select the contour type as the manual and the angel of rotation as 30°. Then 6 slices images were obtained to manually trace the contour lines of the tumor. Once outlining was finished, the 3DUS volume could be obtained automatically. For each tumor, the volume was measured twice to obtain a mean 3DUS volume of each observer.

(4) After the two observers finished their measurements, a total of six measurements were obtained for each tumor. The means of volume by each measurement modality were calculated on the means of the two observers. The measurement time of each modality were also recorded. The measurement time of 2DUS measurement was defined from the 2DUS evaluation to the calculation of 2DUS volume.

The measurement time of 3DUS measurement was defined from 3DUS mode activation to the 3DUS volume obtained by VOCAL method.

### Statistical analysis

Statistical analysis was performed using the SPSS statistical software(Version 25.0) and GraphPad Prism(Version 8.0.0) software. A difference with *P* < 0.05 was considered as statistically significant. Normally distributed continuous variables are expressed as mean ± standard deviation and compared using the paired-samples t-test. Categorical variables were presented as numbers with percentages.

The inter-observer reliability was assessed using ICC with 95% confidence intervals(CIs) based on the absolute agreement and two-way random effects model. Reliability was classified as follows: excellent(ICC > 0.90), good(ICC = 0.75–0.90), moderate(ICC = 0.5–0.74), and poor (ICC < 0.50) [32]. The inter-observer agreement was assessed using Bland-Altman analysis. Agreement was expressed as a bias with 95% limits-of-agreement (LOA). The bias was the tendency for one modality to underestimate or overestimate the measurement relative to the other [33]. LOA was the range within which 95% of the differences between measurements by the two observers or modalities would lie [34], and expressed the absolute magnitude of the agreement between the two observers or modalities. The width of LOA varied with the precision of measurements. LOA was wider when measurements were imprecise and vice versa [35]. The Kolmogorov-Smirnov test was used to assess the normality of the distribution before Bland-Altman analysis, and the measurements were performed as the ratio by the two observers or modalities. The conclusion on agreement should be made based on the width of LOA in comparison to *a priori* defined clinical criteria [35, 36]. According to the 2015 American Thyroid Association(ATA) Guidelines [3], volume changes of less than 50% should be considered as the measurement variation. Therefore, the acceptable agreement of volume in this study should be a LOA ranged from 0.5 to 1.5.

## Results

**Fig. 2:**
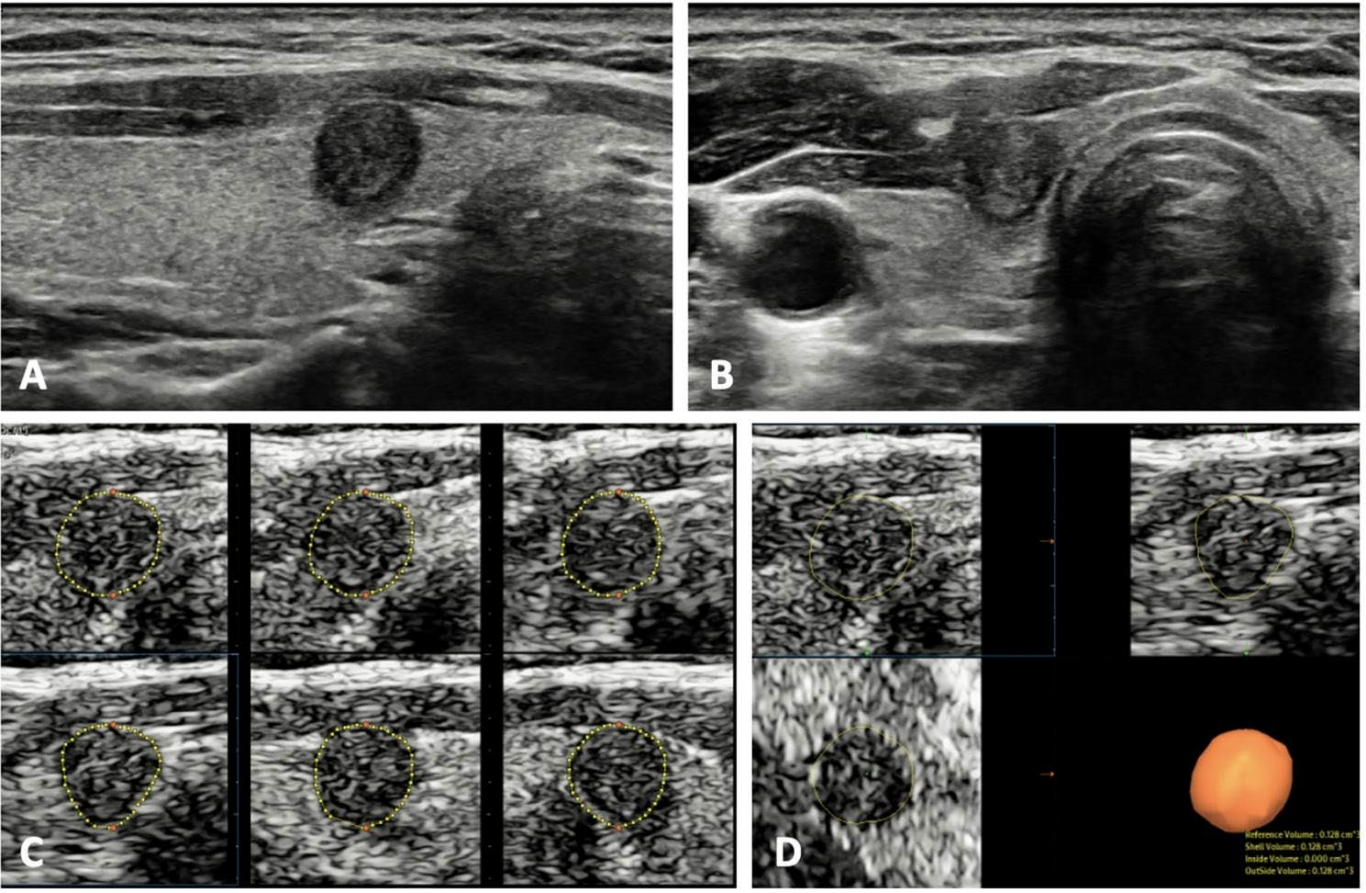
The 2DUS and 3DUS images of a 51-year-old female with PTMC. **A.B.** The longitudinal and transverse images of 2DUS showed a solid tumor located in the right lobe of thyroid. The tumor size was 0.68cmÍ0.65cmÍ0.65 cm and the 2DUS volume was 0.150 cm^3^. **C.** In the measurements of 3DUS volume, the longitudinal US image plane was selected as A plane, and a total of six slices images were obtained to manually trace the contour lines of the tumor. **D.** After the outlining, volume could be obtained automatically with three orthogonal slices simultaneously displayed, and the 3DUS volume was 0.128 cm^3^

**Fig. 3:**
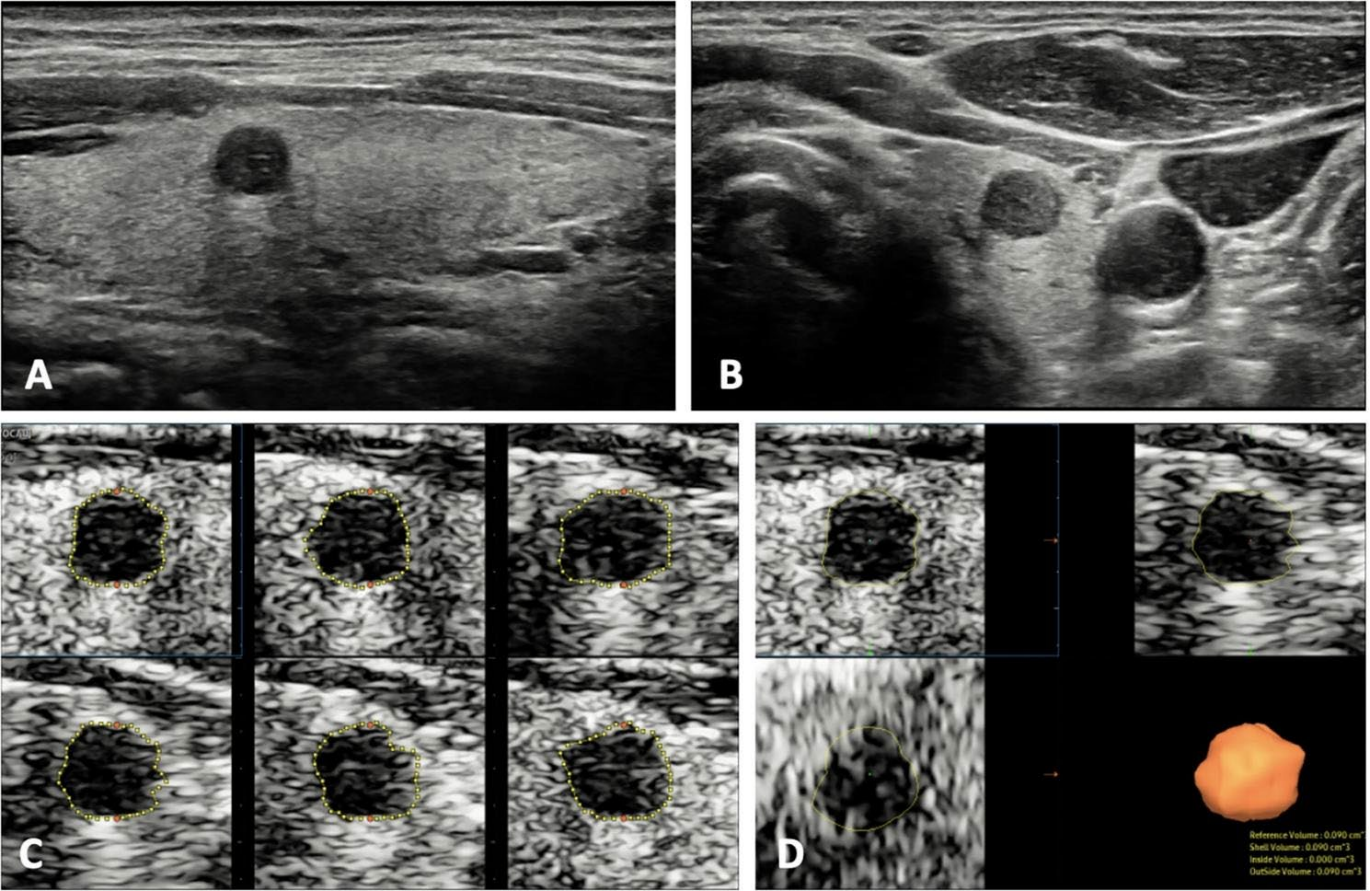
The 2DUS and 3DUS images of a 36-year-old male with PTMC. **A.B.** The longitudinal and transverse images of 2DUS showed a solid tumor located in the left lobe of thyroid. The tumor size was 0.61cmÍ0.57cmÍ0.52 cm and the 2DUS volume was 0.095 cm^3^. **C.** In the measurements of 3DUS volume, the longitudinal US image plane was selected as A plane, and a total of six slices images were obtained to manually trace the contour lines of the tumor. **D.** After the outlining, volume could be obtained automatically with three orthogonal slices simultaneously displayed, and the 3DUS volume was 0.090 cm^3^

A total of 51 patients (46 females, 5 males) with solitary PTMC were included in this study(Table 1). The measurements of PTMC by the two observers are summarized in Table 2. The mean of maximum diameter by two observers was 0.78 ± 0.14 cm. The mean of 2DUS volume and 3DUS volume by two observers was 0.175 ± 0.078 cm^3^ and 0.163 ± 0.074 cm^3^(*P* = 0.005). Representative cases are shown in Figs. 2 and 3.

**Table 1:** Clinical characteristics of patients with PTMC

Characteristics	Data
Age, years	44.3 ± 12.3
Sex	
Female	46(90.2)
Male	5(9.8)
Thyroid parenchyma background	
Normal	42(82.4)
Hashimoto thyroiditis	9(17.6)
Location	
Right lobe	30(58.8)
Left lobe	19(37.3)
Isthmus	2(3.9)
Composition	
Solid	48(94.1)
Mixed cystic and solid	3(5.9)
Echogenicity	
Hyperechoic	1(1.9)
Isoechoic	6(11.8)
Hypoechoic	44(86.3)
Shape	
Taller-than-wide	28(54.9)
Wider-than-tall	23(45.1)
Margin	
Smooth	6(11.8)
Ill-defined	22(43.1)
Lobulated or irregular	23(45.1)
Echogenic Foci	
None	25(49.0)
Macrocalcifications	2(3.9)
Punctate echogenic Foci	24(47.1)

Data are presented as mean ± standard deviation or number of tumors(percentages)

PTMC, papillary thyroid microcarcinoma

**Table 2:** The measurements of PTMC by the two observers

	Observer A	Observer B	*P* value	Mean
Maximum diameter, cm	0.77 ± 0.15	0.78 ± 0.15	0.226	0.78 ± 0.14
2DUS volume, cm^3^	0.175 ± 0.083	0.175 ± 0.078	0.902	0.175 ± 0.078
3DUS volume, cm^3^	0.164 ± 0.076	0.162 ± 0.074	0.659	0.163 ± 0.074

Data are presented as mean ± standard deviation

PTMC, papillary thyroid microcarcinoma; 2DUS, two-dimensional ultrasound; 3DUS, three-dimensional ultrasound

The measurement time of maximum diameter was 54.7 ± 4.8s. The measurement time of 3DUS volume was significantly longer than that of 2DUS (918.85 ± 9.98 s vs. 424.35 ± 9.88 s, *P* < 0.001). The intra-observer reliability and agreement by each observer are shown in supplement table 1.

### Inter-observer reliability

The inter-observer reliability of PTMC measurement were all excellent. The ICCs of inter-observer reliability of maximum diameter, 2DUS volume and 3DUS volume were 0.922(0.864–0.955), 0.928(0.874–0.959), and 0.974(0.955–0.985), respectively. The ICC of inter-observer reliability of volume by two modalities was 0.955(0.909–0.976).

### Inter-observer agreement

**Fig. 4:**
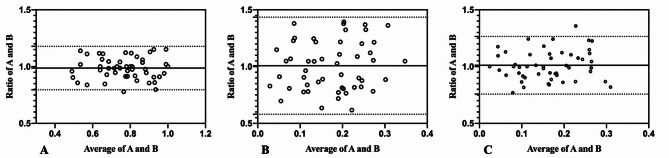
Bland-Altman plots of PTMC measurement by the two observers. **A.** Maximum diameter; **B.** 2DUS volume; C.3DUS volume; The x-axes showed the average of measurements by the two observers. The y-axes showed the ratio between the two observers. Solid lines were the ratio(bias). Top and bottom dashed lines correspond to upper and lower margins of 95% limits-of-agreement(LOA)

**Fig. 5:**
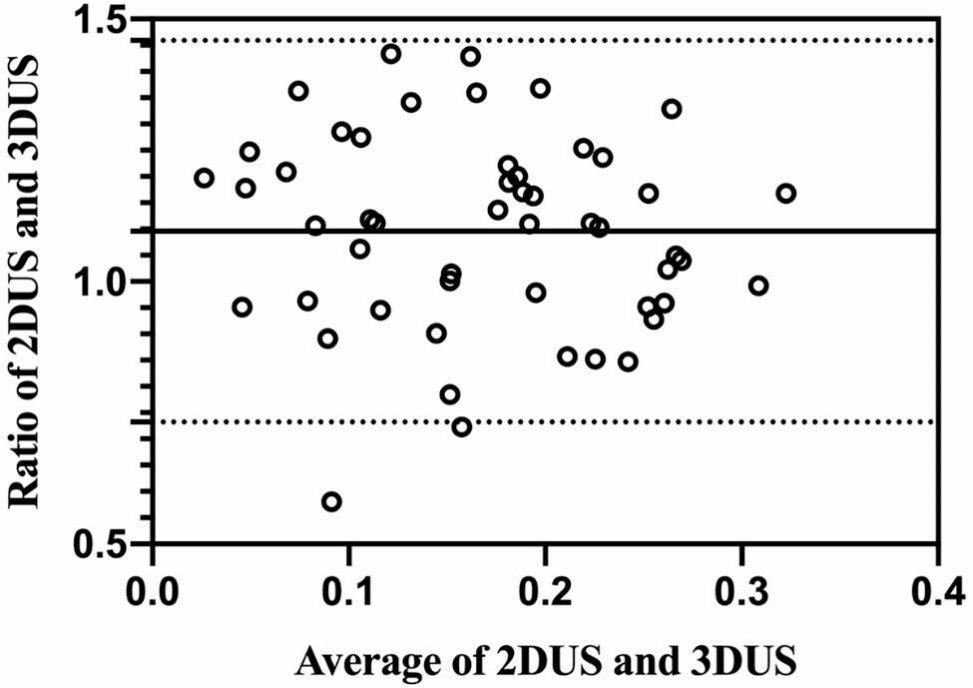
Bland-Altman plots of volume measured by 2DUS and 3DUS. The x-axes showed the average of measurements by the two modalities. The y-axes showed the ratio between the two modalities. Solid lines were the ratio(bias). Top and bottom dashed lines correspond to upper and lower margins of 95% limits-of-agreement(LOA)

The inter-observer agreement of PTMC measurements are summarized in Table 3. The Bland-Altman analysis showed that the bias and 95%LOA of maximum diameter was 0.9869(0.7956–1.178). It means that for about 95% of cases, maximum diameter measured by observer A was between 0.7956 and 1.178 times the maximum diameter measured by observer B. This applied to all the reported LOA hereinafter with corresponding variation. The inter-observer agreement of 2DUS volume and 3DUS volume was 1.008(0.5802–1.435), and 1.011(0.7576–1.265), respectively. The width of 95%LOA of maximum diameter, 2DUS volume and 3DUS volume was 0.3824, 0.8548 and 0.5074. For inter-observer agreement of volume measured by 2DUS and 3DUS, the bias was 1.096, which was above one, and the 95%LOA was from 0.7322 to 1.459. The Bland-Altman plots of PTMC measurements are shown in Figs. 4 and 5.

**Table 3:** The inter-observer agreement of 2DUS and 3DUS in measuring PTMC

	Bias (Ratio)	LOA (width)
Maximum diameter	0.9869	0.7956–1.178(0.3824)
2DUS volume	1.008	0.5802–1.435(0.8548)
3DUS volume	1.011	0.7576–1.265(0.5074)
Volume by 2DUS and 3DUS	1.096	0.7322–1.459(0.7268)

PTMC, papillary thyroid microcarcinoma; 2DUS, two-dimensional ultrasound; 3DUS, three-dimensional ultrasound

## Discussion

It was very important to obtain a reliable measurement of PTMC, as it could be the indication of conversion surgery during AS [5]. This prospective study found that the inter-observer reliability of PTMC measurements were all excellent. The inter-observer agreement (bias and 95%LOA) of maximum diameter, 2DUS volume and 3DUS volume was 0.9869(0.7956–1.178), 1.008(0.5802–1.435), and 1.011(0.7576–1.265), respectively. According to our results, for PTMC, any ratio difference from 0.7322 to 1.459 in maximum diameter, or from 0.5802 to 1.435 in 2DUS volume, or from 0.7576 to 1.265 in 3DUS volume could be considered as the measurement variation for patients with PTMC. Among all the measurements, maximum diameter had the narrowest width of 95% LOA, suggesting maximum diameter had a lowest degree of observer variation for PTMC measurement. Moreover, compared with 2DUS volume, 3DUS volume was significantly smaller and had a narrower 95% LOA. It suggested that volume measured by 3DUS had lower variability and higher repeatability than that obtained by 2DUS.

Because of the indolent nature and favorable outcomes, AS has been recommended as a new management option to immediate surgery for patients with low-risk PTMC [3, 5]. During AS, the evaluation of tumor size enlargement was particularly important, as it could affect the treatment decision-making [5]. Tumor enlargement was defined as growth of more than 3 mm in the maximum diameter or a volume increase greater than 50% [5]. Over 5-year AS, the incidence of volume increase greater than 50% was 24.8–47.5% and of growth of 3.0 mm or more was 12.1–22.4% [12, 14]. However, the observer dependence and measurement variation of US for PTMC have not been considered to determine the meaningful changes in tumor size [17].

To our best knowledge, only one study evaluated the inter-observer variation of 2DUS measurement of PTMC, and the results showed that the 95%LOA of maximum diameter was from − 26.6 to 24.5%, and of volume was from − 65.8 to 64.4%, respectively [19]. It suggested that the inter-observer variation of maximum diameter was smaller than that of volume, which was also consistent with previous studies about measurement variation for well-defined nodule by 2DUS [15, 17, 18]. Similar results were also found in this study. It was because that volume measured by 2DUS was subject to a high degree of observer variation by multiplied in three diameters using the ellipsoid formula method, an increase in volume was more likely to detected than a small increases in diameter [14, 15].

This study also evaluated the inter-observer variation of volume measured by 3DUS. The results found that the inter-observer of 3DUS volume were excellent, and the 95%LOA was from 0.7576 to 1.265. It indicated that different observers did not affect the measurement, and 3DUS could be a reliable and reproducible volume measurement of PTMC. Moreover, the bias of volume measured by 2DUS and 3DUS was above one. Compared with 2DUS volume, 3DUS volume were significantly smaller and had a narrower 95% LOA. It suggested that volume measured by 2DUS was overestimated, and volume measured by 3DUS was more reliable than those obtained by 2DUS, which was consistent with previous studies [21, 25, 26]. These results can be explained by the different measurement methods of the two modalities. 2DUS used the ellipsoid formula method to calculate the volume, which was based on the assumption that the object was an ellipse [15]. However, for PTMC that usually had an ill-defined or irregular margin rather than a smooth one, this method could be overestimated the volume when multiplied by three diameters in the calculation. As a result, volume of irregular shaped tumor measured by 2DUS could have a high inter-observer variation [25, 26]. In contrast, these deficiencies could be avoided when volume was measured using 3DUS, which had a significant potential for increasing the reproducibility of volume measurement [20, 28]. Because 3DUS could easily obtain multiple slice images which encompassed the entire tumor, the border of tumor could be sensitively detected and manually outlined to calculate the final volume, even if the target object was small [21].

Although 3DUS could accurately reflect changes in tumor size and identify of tumor enlargement to inform the timing of conversion surgery during AS, there were drawbacks that limit its clinical application in routine evaluation. First, the borders identification by 3DUS could be subject to the errors because not all the slices had high imaging quality as well as 2DUS. Although this study showed that the 95% LOA of 3DUS volume was within the clinical criteria, the measurement still needed to be cautious. Second, 3DUS required additional processing of manually outlining the tumor border after scanning, leading to labor-intensive and time-consuming. Third, compared with maximum diameter, the 95%LOAof 3DUS was still relatively larger. It suggested that maximum diameter was not only a practical and simple method for tumor enlargement, but also had high reproducibility. However, the tumor growth pattern of PTMC was complicated. Some studies reported that PTMC grew rather rapidly in the initial stages of progression, and in many cases the growth decreased or even vanished at certain time points [13, 37]. Therefore, a comprehensive evaluation of tumor size during AS is needed and 3DUS could provide more reliable estimates of tumor volume change.

This study had limitations. First, this study only evaluated the inter-observer measurement variation of PTMC, thus we did not obtain the true volume of tumor. Second, only one US machine has been used in this study. In clinical routine procedure, it was almost impossible to measure the tumor by the same US machine at each follow-up period. Further studies used two different machines are needed to confirm the results. Third, because only 9 patients in this study had Hashimoto thyroiditis, its impact on the measurement variation was not evaluated. Fourth, this study only enrolled patients with solitary PTMC. Further study is needed to investigate whether the results can be applied to multifocal PTMC.

## Conclusions

The inter-observer reliability of PTMC measurements by 2DUS and 3DUS were excellent. For PTMC, any ratio difference from 0.7322 to 1.459 in maximum diameter, or from 0.5802 to 1.435 in 2DUS volume, or from 0.7576 to 1.265 in 3DUS volume could be considered as the measurement variation for patients with PTMC. Maximum diameter had the lowest degree of observer variation, which was more practical and simple measurement of PTMC. Volume measured by 3DUS had lower variability and higher repeatability than that by 2DUS, which might be helpful to provide more reliable estimates of tumor size for PTMC.

### Electronic supplementary material

Below is the link to the electronic supplementary material.

Supplementary Material 1

## Data Availability

The datasets used and/or analyzed during the current study are available from the corresponding author on reasonable request.

## References

[1] Sung H, Ferlay J, Siegel RL, Laversanne M, Soerjomataram I, Jemal A (2021). Global Cancer Statistics 2020: GLOBOCAN estimates of incidence and Mortality Worldwide for 36 cancers in 185 countries. CA Cancer J Clin.

[2] Cronin KA, Scott S, Firth AU, Sung H, Henley SJ, Sherman RL (2022). Annual report to the nation on the status of cancer, part 1: National cancer statistics. Cancer.

[3] Haugen BR, Alexander EK, Bible KC, Doherty GM, Mandel SJ, Nikiforov YE (2016). 2015 american thyroid Association Management Guidelines for adult patients with thyroid nodules and differentiated thyroid Cancer: the american thyroid Association Guidelines Task Force on thyroid nodules and differentiated thyroid Cancer. Thyroid.

[4] Sanabria A, Betancourt-Aguero C, Sanchez-Delgado JG, Garcia-Lozano C (2022). Prophylactic Central Neck Lymph Node Dissection in low-risk thyroid carcinoma patients does not decrease the incidence of Locoregional recurrence: a Meta-analysis of Randomized trials. Ann Surg.

[5] Sugitani I, Ito Y, Takeuchi D, Nakayama H, Masaki C, Shindo H (2021). Indications and strategy for active surveillance of adult low-risk papillary thyroid Microcarcinoma: Consensus statements from the Japan Association of endocrine surgery Task Force on Management for Papillary thyroid Microcarcinoma. Thyroid.

[6] Hwangbo Y, Choi JY, Lee EK, Ryu CH, Cho SW, Chung EJ (2022). A cross-sectional survey of patient treatment choice in a Multicenter prospective cohort study on active surveillance of papillary thyroid microcarcinoma (MAeSTro). Thyroid.

[7] Chou R, Dana T, Haymart M, Leung AM, Tufano RP, Sosa JA (2022). Active Surveillance Versus thyroid surgery for differentiated thyroid Cancer: a systematic review. Thyroid.

[8] Ito Y, Uruno T, Nakano K, Takamura Y, Miya A, Kobayashi K (2003). An observation trial without surgical treatment in patients with papillary microcarcinoma of the thyroid. Thyroid.

[9] Cho SJ, Suh CH, Baek JH, Chung SR, Choi YJ, Chung KW (2019). Active surveillance for small papillary thyroid Cancer: a systematic review and Meta-analysis. Thyroid.

[10] Hwang H, Choi JY, Yu HW, Moon JH, Kim JH, Lee EK et al. Surgical Outcomes in patients with low-risk papillary thyroid Microcarcinoma from MAeSTro Study: Immediate Operation Versus delayed operation following active surveillance a multicenter prospective cohort study. Ann Surg. 2023. 10.1097/SLA.0000000000005841 36912439

[11] Ito Y, Miyauchi A, Inoue H, Fukushima M, Kihara M, Higashiyama T (2010). An observational trial for papillary thyroid microcarcinoma in japanese patients. World J Surg.

[12] Tuttle RM, Fagin JA, Minkowitz G, Wong RJ, Roman B, Patel S (2017). Natural history and tumor volume kinetics of papillary thyroid cancers during active surveillance. JAMA Otolaryngol Head Neck Surg.

[13] Ito Y, Miyauchi A, Kudo T, Higashiyama T, Masuoka H, Kihara M (2019). Kinetic analysis of growth activity in enlarging papillary thyroid Microcarcinomas. Thyroid.

[14] Oh HS, Ha J, Kim HI, Kim TH, Kim WG, Lim DJ (2018). Active surveillance of low-risk papillary thyroid microcarcinoma: a Multi-Center Cohort Study in Korea. Thyroid.

[15] Brauer VFH, Eder P, Miehle K, Wiesner TD, Hasenclever H, Paschke R (2005). Interobserver variation for ultrasound determination of thyroid nodule volumes. Thyroid.

[16] Haser GC, Tuttle RM, Su HK, Alon EE, Bergman D, Bernet V (2016). Active surveillance for papillary thyroid Microcarcinoma: New Challenges and Opportunities for the Health Care System. Endocr Pract.

[17] Lee HJ, Yoon DY, Seo YL, Kim JH, Baek S, Lim KJ (2018). Intraobserver and Interobserver Variability in Ultrasound measurements of thyroid nodules. J Ultrasound Med.

[18] Choi YJ, Baek JH, Hong MJ, Lee JH (2015). Inter-Observer Variation in Ultrasound Measurement of the volume and diameter of thyroid nodules. Korean J Radiol.

[19] Chung SR, Choi YJ, Lee SS, Kim SO, Lee SA, Jeon MJ (2021). Interobserver Reproducibility in Sonographic Measurement of Diameter and volume of papillary thyroid microcarcinoma. Thyroid.

[20] Kim SC, Kim JH, Choi SH, Yun TJ, Wi JY, Kim SA (2016). Off-site evaluation of three-dimensional ultrasound for the diagnosis of thyroid nodules: comparison with two-dimensional ultrasound. Eur Radiol.

[21] You JH, Zhuang YF, Lu MZ, Chen L, Chen ZK, Chen XK (2020). Three–Dimensional Ultrasonography in Preoperative and postoperative volume Assessment of the undescended testicle. Med Sci Monit.

[22] Czuczwar P, Wozniak S, Szkodziak P, Milart P, Wozniakowska E, Wrona W (2015). Influence of ulipristal acetate therapy compared with uterine artery embolization on fibroid volume and vascularity indices assessed by three-dimensional ultrasound: prospective observational study. Ultrasound Obstet Gynecol.

[23] Boers T, Braak SJ, Versluis M, Manohar S (2021). Matrix 3D ultrasound-assisted thyroid nodule volume estimation and radiofrequency ablation: a phantom study. Eur Radiol Exp.

[24] Ying M, Pang BS (2009). Three-dimensional ultrasound measurement of cervical lymph node volume. Br J Radiol.

[25] Schlögl S, Werner E, Lassmann M, Terekhova J, Muffert S, Seybold S (2001). The use of three-dimensional ultrasound for thyroid volumetry. Thyroid.

[26] Lyshchik A, Drozd V, Schloegl S, Reiners C (2004). Three-dimensional ultrasonography for volume measurement of thyroid nodules in children. J Ultrasound Med.

[27] Lyshchik A, Drozd V, Reiners C (2004). Accuracy of three-dimensional ultrasound for thyroid volume measurement in children and adolescents. Thyroid.

[28] Freesmeyer M, Wiegand S, Schierz JH, Winkens T, Licht K (2014). Multimodal evaluation of 2-D and 3-D ultrasound, computed tomography and magnetic resonance imaging in measurements of the thyroid volume using universally applicable cross-sectional imaging software: a phantom study. Ultrasound Med Biol.

[29] Lagendijk M, Vos EL, Ramlakhan KP, Verhoef C, Koning AHJ, van Lankeren W (2018). Breast and tumour volume measurements in breast Cancer patients using 3-D automated breast volume scanner images. World J Surg.

[30] Tessler FN, Middleton WD, Grant EG, Hoang JK, Berland LL, Teefey SA (2017). ACR thyroid imaging, reporting and Data System (TI-RADS): White Paper of the ACR TI-RADS Committee. J Am Coll Radiol.

[31] Frates MC, Benson CB, Charboneau JW, Cibas ES, Clark OH, Coleman BG (2005). Management of thyroid nodules detected at US: Society of Radiologists in Ultrasound consensus conference statement. Radiology.

[32] Koo TK, Li MY (2016). A Guideline of selecting and reporting Intraclass correlation coefficients for Reliability Research. J Chiropr Med.

[33] Anvari A, Halpern EF, Samir AE (2018). Essentials of statistical methods for assessing reliability and agreement in quantitative imaging. Acad Radiol.

[34] Bland JM, Altman DG (1999). Measuring agreement in method comparison studies. Stat Methods Med Res.

[35] Chhapola V, Kanwal SK, Brar R (2015). Reporting standards for Bland-Altman agreement analysis in laboratory research: a cross-sectional survey of current practice. Ann Clin Biochem.

[36] Mantha S, Roizen MF, Fleisher LA, Thisted R, Foss J (2000). Comparing methods of clinical measurement: reporting standards for bland and altman analysis. Anesth Analg.

[37] Miyauchi A, Kudo T, Ito Y, Oda H, Yamamoto M, Sasai H (2019). Natural history of papillary thyroid microcarcinoma: kinetic analyses on tumor volume during active surveillance and before presentation. Surgery.

