# Supplementary material for: Inter-observer variation in two-dimensional and three-dimensional ultrasound measurement of papillary thyroid microcarcinoma
Source: Cancer Imaging. 2023 Oct 5;23:94. doi: 10.1186/s40644-023-00613-3 (PMC10557328; doi:10.1186/s40644-023-00613-3)
Supplement: Supplementary file 1 — Supplementary Material 1 [file 40644_2023_613_MOESM1_ESM.docx]

Supplement Table. The intra-observer reliability and agreement of 2DUS and 3DUS

|  | ICCs | | Agreement(bias with LOA) | |
| --- | --- | --- | --- | --- |
|  | Observer A | Observer B | Observer A | Observer B |
| 2DUS volume | 0.994(0.884-0.970) | 0.947(0.908-0.970) | 0.9367(0.5370-1.336) | 1.052(0.6311-1.473) |
| 3DUS volume | 0.982(0.969-0.990) | 0.958(0.927-0.976) | 0.991(0.7695-1.212) | 1.039(0.6686-1.408) |

ICC, intraclass correlation coefficient; LOA, limits of agreement; 2DUS, two-dimensional ultrasound; 3DUS, three-dimensional ultrasound
